# Retargeted Foamy Virus Vectors Integrate Less Frequently Near Proto-oncogenes

**DOI:** 10.1038/srep36610

**Published:** 2016-11-04

**Authors:** Jonah D. Hocum, Ian Linde, Dustin T. Rae, Casey P. Collins, Lindsay K. Matern, Grant D. Trobridge

**Affiliations:** 1 Department of Pharmaceutical Sciences, Washington State University, Spokane, WA, 99210, United Sates; 2 School of Molecular Biosciences, Washington State University, Pullman, WA, 99164, United Sates

## Abstract

Retroviral gene therapy offers immense potential to treat many genetic diseases and has already shown efficacy in clinical trials. However, retroviral vector mediated genotoxicity remains a major challenge and clinically relevant approaches to reduce integration near genes and proto-oncogenes are needed. Foamy retroviral vectors have several advantages over gammaretroviral and lentiviral vectors including a potentially safer integration profile and a lower propensity to activate nearby genes. Here we successfully retargeted foamy retroviral vectors away from genes and into satellite regions enriched for trimethylated histone H3 at lysine 9 by modifying the foamy virus Gag and Pol proteins. Retargeted foamy retroviral vectors integrated near genes and proto-oncogenes less often (*p* < 0.001) than controls. Importantly, retargeted foamy retroviral vectors can be produced at high, clinically relevant titers (>10^7^ transducing units/ml), and unlike other reported retargeting approaches engineered target cells are not needed to achieve retargeting. As proof of principle for use in the clinic we show efficient transduction and retargeting in human cord blood CD34^+^ cells. The modified Gag and Pol helper constructs we describe will allow any investigator to simply use these helper plasmids during vector production to retarget therapeutic foamy retroviral vectors.

Retroviral vector gene therapy offers immense potential to treat many genetic diseases^1^ and has shown efficacy in clinical trials^2^,^3^,^4^. The joint SCID-X1 trial between Paris and London demonstrated the success of retroviral vector gene therapy with 17 of 20 patients successfully treated. However, 5 patients developed leukemia as an adverse side effect from the retroviral vector^5^,^6^. Vector mediated genotoxicity results when the integrated vector dysregulates or disrupts host genes which can lead to oncogenic transformation^7^. Despite these risks gene therapy remains a promising alternative to allogeneic transplantation^8^. However, safer vector systems are needed to reduce the risk of genotoxicity.

The integration profile of different retroviral vectors have been defined to assess their relative safety^9^,^10^,^11^,^12^. These studies have shown that different vector types have different integration profiles and that the gammaretroviruses used in the SCID-X1 trials preferentially integrate near gene promoters. Significant efforts have been made to reduce the genotoxic potential of retroviral vectors. Self-inactivating (SIN) vectors have reduced genotoxic potential due to the lack of enhancers in the LTRs^13^, but they still have the ability to dysregulate or disrupt transcription of host genes by other mechanisms, such as increased transcription of host genes from an internal vector promoter or insertional inactivation^7^. Thus, vectors that integrate farther away from genes may be safer for clinical use. The viral pre-integration complex (PIC) contributes to the integration profile of viral vectors by interacting with host cell and viral proteins^14^,^15^. Retroviral PICs are poorly defined but include the vector genome and viral proteins including the viral integrase (IN) which catalyzes integration within the host genome^16^.

Redirecting vectors to safe locations for integration has potential to increase the safety of retroviral vectors in gene therapy approaches. The lentiviral PIC is known to interact with lens epithelium-derived growth factor (LEDGF), which plays a major role in directing lentiviral integration to actively transcribed genes^17^,^18^,^19^. Gijsbers *et al.* showed that lentiviral vector (LV) integration can be retargeted towards gene-poor regions in the genome by modifying LEDGF^20^. In their approach, host cell lines were modified to express chromobox protein homolog 1 (CBX1) fused to LEDGF. CBX1 interacts with tri-methylated lysine 9 of histone H3 (H3K9me3) and is associated with gene sparse regions in the human genome^21^. Although this approach was successful in retargeting LV integration, it requires modifying a target cell line to express a chimeric LEDGF-CBX1 protein, which is not practical for clinical use.

Foamy retroviral vectors (FVs) have several desirable properties in regards to genotoxicity. In contrast to gammaretroviral vectors (GVs), FVs naturally have a lower tendency to integrate within CpG islands and near promoters^12^. FVs also integrate less often within genes than LVs. In addition, FVs have not been shown to exhibit read-through transcription and have a lower potential to activate nearby genes than either GVs or LVs^22^. The interactions of the foamy retroviral PIC with host proteins are not as well defined as lentiviral PIC interactions. However, the foamy virus Gag contains a chromatin binding site (CBS) motif in the C-terminus. The CBS is present in foamy retroviral PICs and interacts with the core histones H2A and H2B on host chromatin^23^. The CBS is a distinguishing feature of the foamy virus Gag protein. Mutations in the CBS negatively affect the ability of the foamy virus PIC to interact with host chromatin.

Here we demonstrate a clinically relevant approach to retarget FV integration into satellite elements and H3K9me3 regions and away from proto-oncogenes. We were able to significantly alter the integration profile of FVs, reducing the frequency of retroviral vector integration sites (RISs) near genes and proto-oncogenes. Efficient retargeting required modifying the FV Gag CBS and fusing the FV IN to CBX1. Importantly, using this approach retargeted FVs can be produced at clinically relevant titers and any target cell, including CD34^+^ human cord blood cells, can be used without prior modification by simply using alternate foamy helper plasmids during vector production.

## Results

### Retargeted FVs can be produced at clinically relevant titers

**Figure 1:**
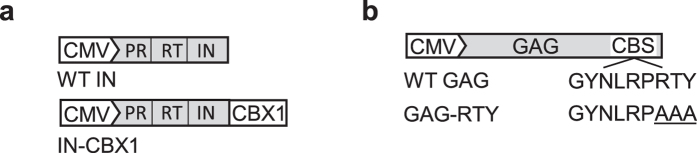
Modified FV Pol and Gag helper plasmids for retargeted integration into heterochromatin. (**a**) Schematic representations of the normal and modified Pol constructs which encode the viral protease (PR), reverse transcriptase (RT), and integrase (IN) proteins. The modified Pol construct expresses chromobox protein homolog 1 (CBX1) fused to IN (IN-CBX1) to retarget integration. (**b**) Schematic representations of the normal and modified Gag constructs. The modified Gag construct expresses a chromatin binding site (CBS) domain with a triple alanine substitution of RTY (Gag-RTY). The triple alanine mutation reduces the binding affinity of CBS with host chromatin. CMV, cytomegalovirus promoter; WT, wild type.

**Figure 2:**
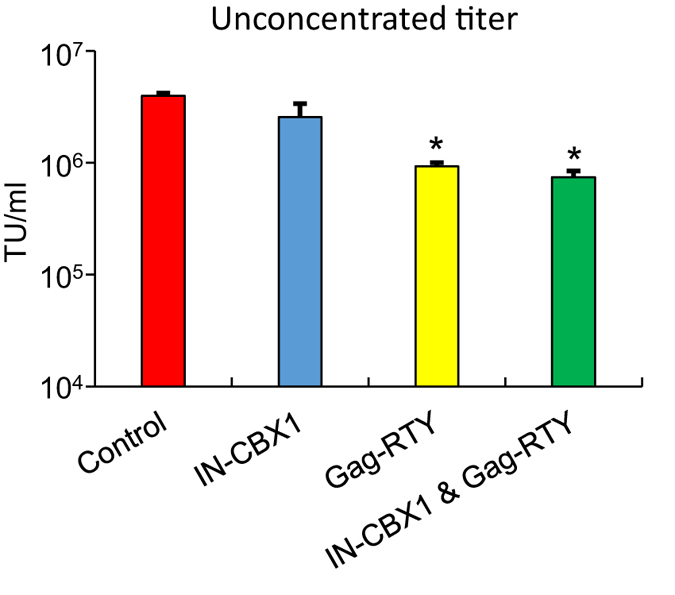
Retargeted FVs can be produced at clinically relevant titers. Unconcentrated titers in triplicate (mean + SEM). *Statistically significant at *p* < 0.05 compared to control vector. TU, transducing units.

To retarget FVs we developed 2 modified FV helper plasmids. The FV Gag helper plasmid CBS was altered using a previously described triple alanine substitution of RTY mutation shown to eliminate chromatin binding^23^. FV Gag (pFVGag-CBS-RTY) expresses a CBS domain with the triple alanine substitution of RTY (Gag-RTY). The FV Pol helper plasmid (pFVPol-CBX1) expresses CBX1 fused to the C-terminus of IN (IN-CBX1) with a flexible glycine-serine, (GS)_3_, linker (Fig. 1a,b). In this approach modified FVs are expected to integrate in areas enriched for H3K9me3 that are not close to genes. A control vector preparation (FV_Control_) and 3 modified vector preparations (FV_IN-CBX1_, FV_Gag-RTY_, and FV_IN-CBX1 & Gag-RTY_) were produced in parallel by transient transfection with the appropriate Pol, Gag, and Env helper plasmids and the FV-SGW-KO vector plasmid, which expresses an enhanced green fluorescent protein (EGFP) gene. Vector titers were determined by exposing HT1080 cells to vector containing media and analyzing the cells for EGFP expression. Although the unconcentrated titers of FV_Gag-RTY_ and FV_IN-CBX1 & Gag-RTY_ were between 4 and 5 fold lower than control unmodified FV (Fig. 2), all modified FVs could be produced at clinically relevant titers, >10^7^ transducing units (TU)/ml, after a 100-fold concentration.

### Retargeted FVs integrate near H3K9me3 enriched regions and satellite elements

**Figure 3:**
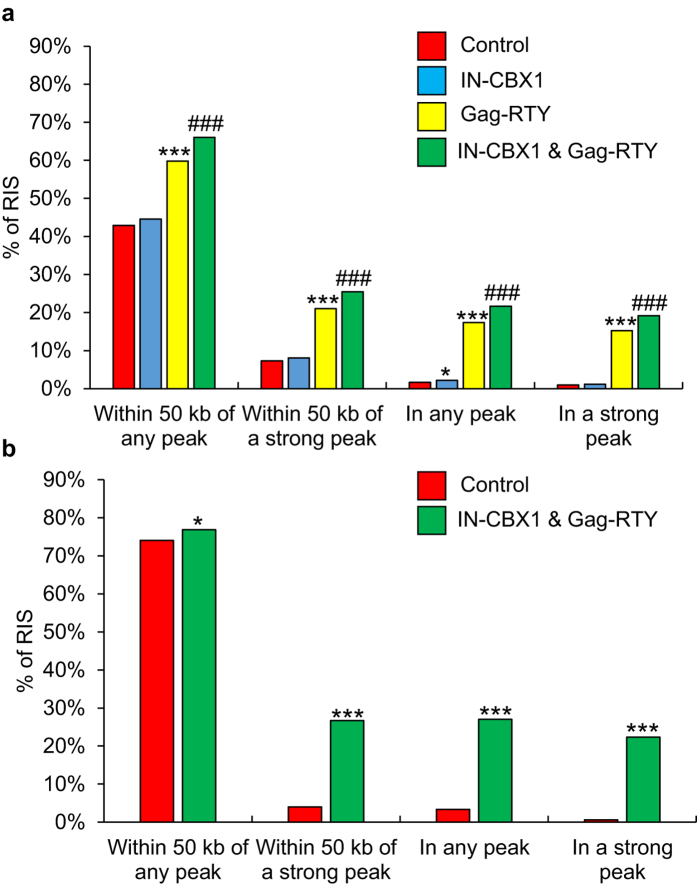
Retargeted FVs have a greater preference for H3K9me3 regions in IMR90 and CD34^+^ cells. (**a**) Observed retargeting in IMR90 cells. (**b**) Observed retargeting in CD34^+^ cells. *Statistically significant at *p* < 0.05 compared to control vector. ***Statistically significant at *p* < 0.001 compared to control vector. ^###^Statistically significant at *p* < 0.001 compared to control, Gag-RTY, and IN-CBX1 vectors. RIS, retroviral vector integration sites.

We next asked whether the modified FVs showed a preference for integrating into or near H3K9me3 histone modified regions since CBX1 is known to bind to H3K9me3. IMR90 normal human fibroblast cells were transduced with the control and modified vectors. RISs were sequenced using modified genome sequencing (MGS)-PCR^24^,^25^, resulting in over 900 captured RISs (Table 1) for each vector. The RISs were analyzed to determine the distance of each site relative to H3K9me3 peaks in IMR90 cells previously determined by ChIP-Seq. FV_IN-CBX1 & Gag-RTY_ was significantly retargeted the most efficiently relative to FV_Control_ (*p* < 0.001), with 19.2% of the RISs being within areas with high levels of H3K9me3 (Fig. 3a). Interestingly, FV_Gag-RTY_ also had a higher number of RISs within H3K9me3 regions when compared to FV_Control_ (*p* < 0.001). FV with only the Pol mutation, FV_IN-CBX1,_ had a weaker but significant retargeting for H3K9me3 regions (*p* < 0.05). Thus, eliminating the Gag CBS binding domain had a stronger effect on retargeting than the IN-CBX1 fusion, but the combination of both mutations led to the strongest retargeting.

**Figure 4:**
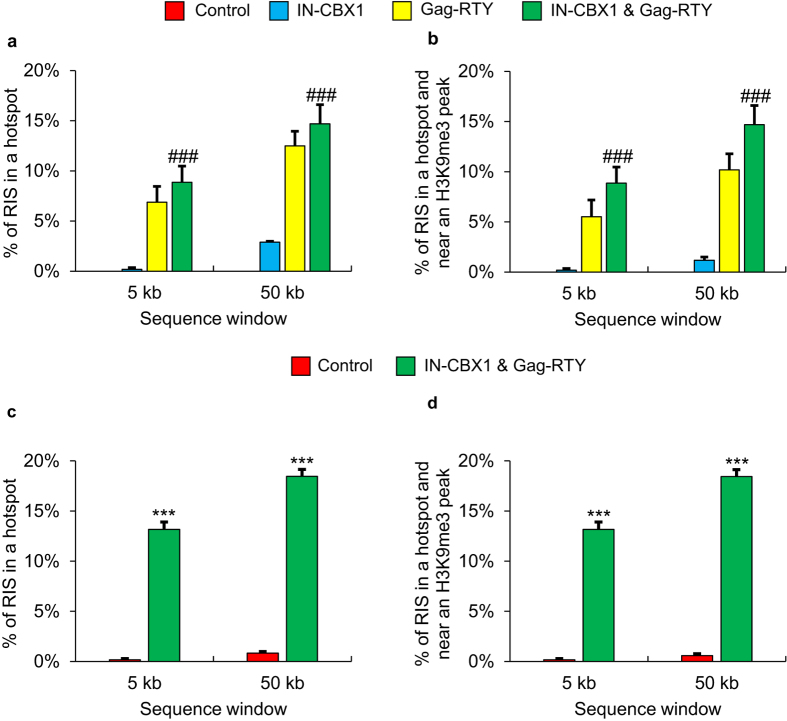
Retargeted FVs form RIS hotspots in H3K9me3 regions. The number of FV RIS hotspots was determined for each vector. Hotspots are defined as 3 RISs within a sequence window (5 kb and 50 kb). RIS data were divided into a minimum of three non-overlapping subsets of (**a**) 320 RISs for IMR90 datasets and (**c**) 511 RISs for CD34^+^ datasets. The mean + SEM is plotted. Then the proximity of the subset of RIS that were in hotspots to H3K9me3 peaks was determined for (**b**) IMR90 cell data and (**d**) CD34^+^ cell data. RISs were considered near an H3K9me3 peak if they were within 50 kb. ***Statistically significant at *p* < 0.001 compared to control vector. ^###^Statistically significant at *p* < 0.001 compared to Gag-RTY and IN-CBX1 vectors. RIS, retroviral vector integration site.

Since the combination of IN-CBX1 and Gag-RTY proteins had the most dramatic effect on retargeting FV integration into H3K9me3 regions, CD34^+^ cells were transduced with the FV_IN-CBX1 & Gag-RTY_ and FV_Control_ to compare RISs in clinically relevant cells. The redistribution of the RISs with the FV_IN-CBX1 & Gag-RTY_ vector in CD34^+^ cells was consistent with the trend observed in IMR90 cells (Fig. 3b). Efficient retargeting of FV_IN-CBX1 & Gag-RTY_ integration to H3K9me3 sites in CD34^+^ cells shows that, as expected, retargeting was not specific to the fibroblast cells. We further assessed the number of RISs that were within hotspots (Fig. 4a,c and Supplemental Figure S1). FV_IN-CBX1 & Gag-RTY_ had a much greater tendency to form RIS hotspots than FV_Control_ in both IMR90 and CD34^+^ cells. Importantly, the vast majority of the RISs that fell within hotspots were also near (<50 kb) H3K9me3 regions (Fig. 4b,d). We also investigated the proximity of the RISs from IMR90 cells to other histone modification and DNase I hypersensitive site data (Supplemental Figure S2). FV_IN-CBX1 & Gag-RTY_ had a significant but slight preference for DNase I hypersensitivity, H3K9ac, and H3K4me3 sites, which are associated with high levels of transcription, when compared to FV_Control_.

We also expected that retargeting away from genes might increase the frequency of integration into repeat elements. Therefore, we analyzed whether the retargeted vectors had a preference for any repeat elements (Table 2). FV_Gag-RTY_ and FV_IN-CBX1 & Gag-RTY_ RISs were retargeted out of LINE, SINE, DNA, and LTR elements and both vectors had significantly more RISs in satellite elements (*p* < 0.001) when compared to FV_Control_.

### Retargeted FVs integrate less frequently near genes and proto-oncogenes

**Figure 5:**
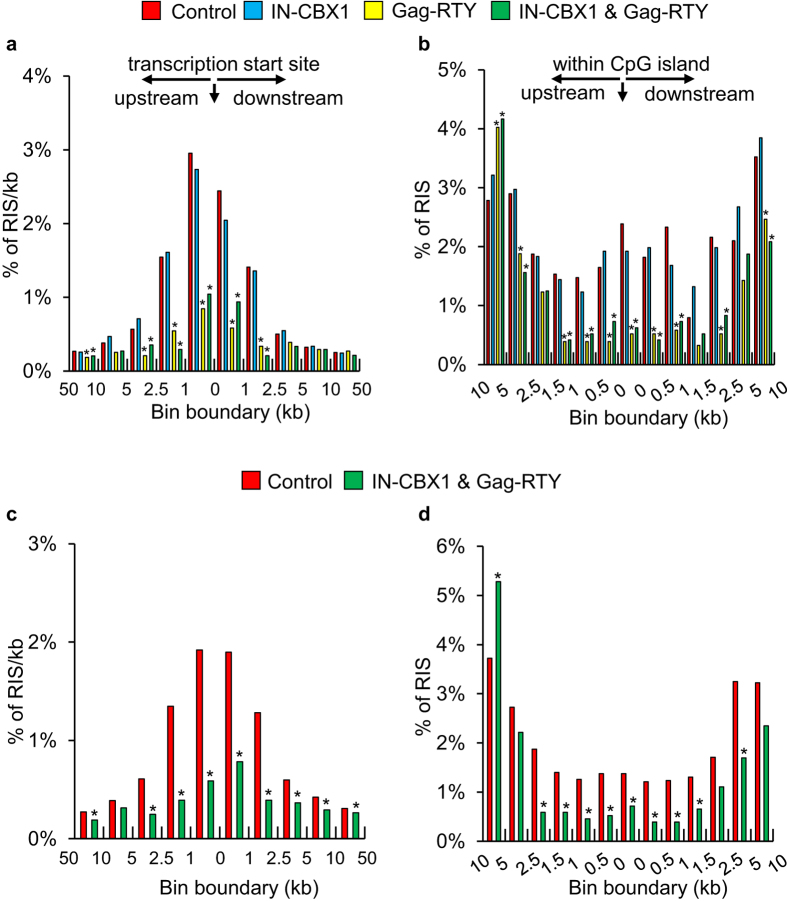
Retargeted FVs integrate farther away from transcription start sites and CpG islands. Proximity of RIS in IMR90 cells to (**a**) transcription start sites and (**b**) CpG islands. Proximity of RIS in CD34^+^ cells to (**c**) transcription start sites and (**d**) CpG islands. *Statistically significant at *p* < 0.05 compared to control vector. RIS, retroviral vector integration sites.

FVs have distinct RIS preferences relative to genes and CpG islands when compared to LVs and GVs^12^. Since a major determinant of retroviral vector safety is the distance of RISs to proto-oncogenes and regulatory elements, the proximity of retargeted RISs to RefSeq genes, CpG islands, and proto-oncogenes was determined in both IMR90 and CD34^+^ cells. Retargeted FVs had distinct and favorable integration patterns from control FV relative to transcription start sites (TSSs) and CpG islands (Fig. 5). There was dramatic retargeting away from TSSs and CpG islands with the FV_Gag-RTY_ and FV_IN-CBX1 & Gag-RTY_ vectors when compared to FV_Control_. Importantly, the number of RISs near proto-oncogene TSSs was significantly reduced (*p* < 0.001) for FV_Gag-RTY_ and FV_IN-CBX1 & Gag-RTY_ retargeted FVs (Table 1) in normal human fibroblasts, and for FV_IN-CBX1 & Gag-RTY_ retargeted FVs in human CD34^+^ cells. Thus, as expected, retargeting integration into H3K9me3 enriched regions and satellite elements reduced the number of RISs near genes and proto-oncogenes (Table 1 and Supplemental Table S1).

### Cells transduced with retargeted FVs have efficient transgene expression and display no evidence of clonal outgrowth

**Figure 6:**
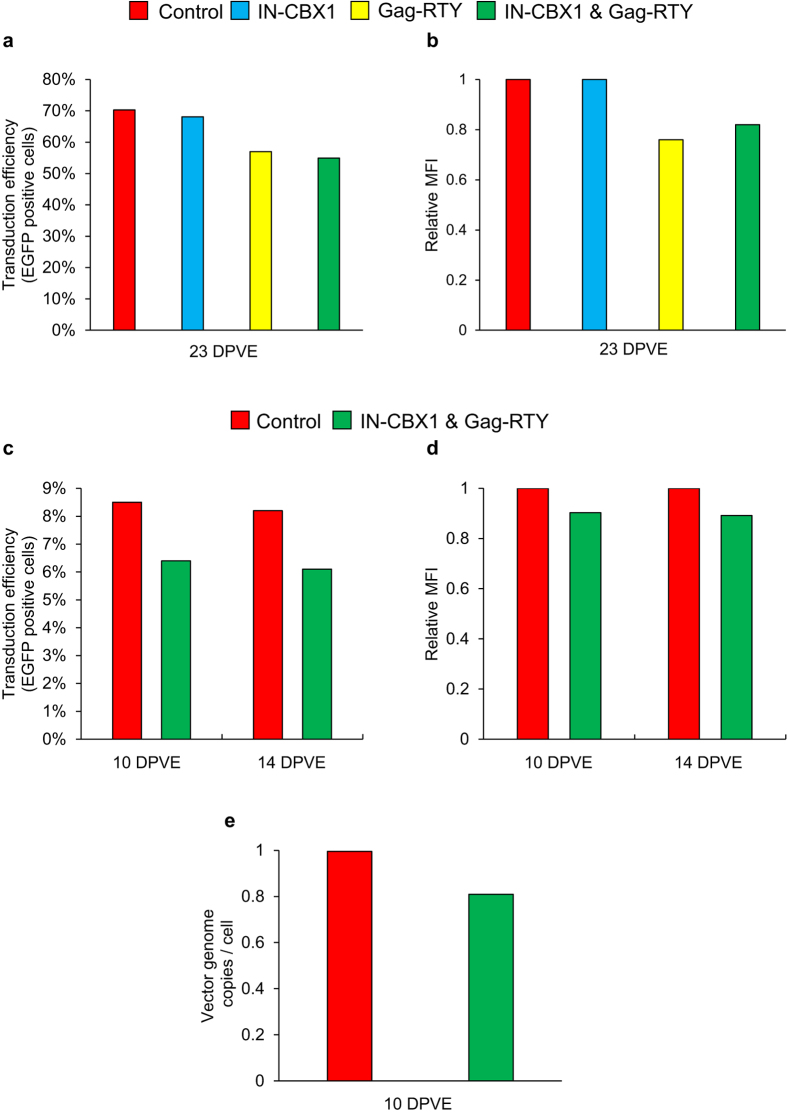
Retargeted FVs mediate a high level of transgene expression with no evidence of vector silencing in human cord blood CD34^+^ cells. Transduction efficiency based on the percent of EGFP positive cells and MFI relative to the control in (**a,b**) IMR90 and (**c,d**) CD34^+^ cell populations. (**e**) Number of vector genome copies based on a quantitative PCR assay observed in CD34^+^ cells. MFI; mean fluorescence intensity.

Since the retargeted FVs integrated in transcriptionally less active regions in the genome, we analyzed the transgene expression in the transduced cells by flow cytometry. EGFP expression was slightly lower for all retargeted vectors when compared to the control in both cell lines, but it is important to note that there was still a relatively high level of EGFP expression, 1.3–1.4 fold lower than the control, for all modified vectors in both normal human fibroblasts at a multiplicity of infection (MOI) of 0.5, and cord blood CD34^+^ cells at a MOI of 10. Vector genome copy numbers from CD34^+^ genomic DNA were determined with quantitative PCR (Fig. 6). FV_IN-CBX1 & Gag-RTY_ had 19% fewer vector copies per cell when compared to FV_Control_, indicating the lower level of EGFP expression was due to fewer vector copies and not evidence of increased silencing for retargeted FV. We additionally performed colony forming unit (CFU) assays to determine if the retargeted FV efficiently transduced hematopoietic progenitor cells (Supplemental Figure S3). The difference in the percentage of EGFP positive CFUs, 12.3% for control FV sample and 9.4% for retargeted FV sample, was similar to the control and retargeted FV bulk CD34^+^ population flow cytometry analysis for EGFP, again showing no evidence of increased silencing, even after 14 days in culture.

**Figure 7:**
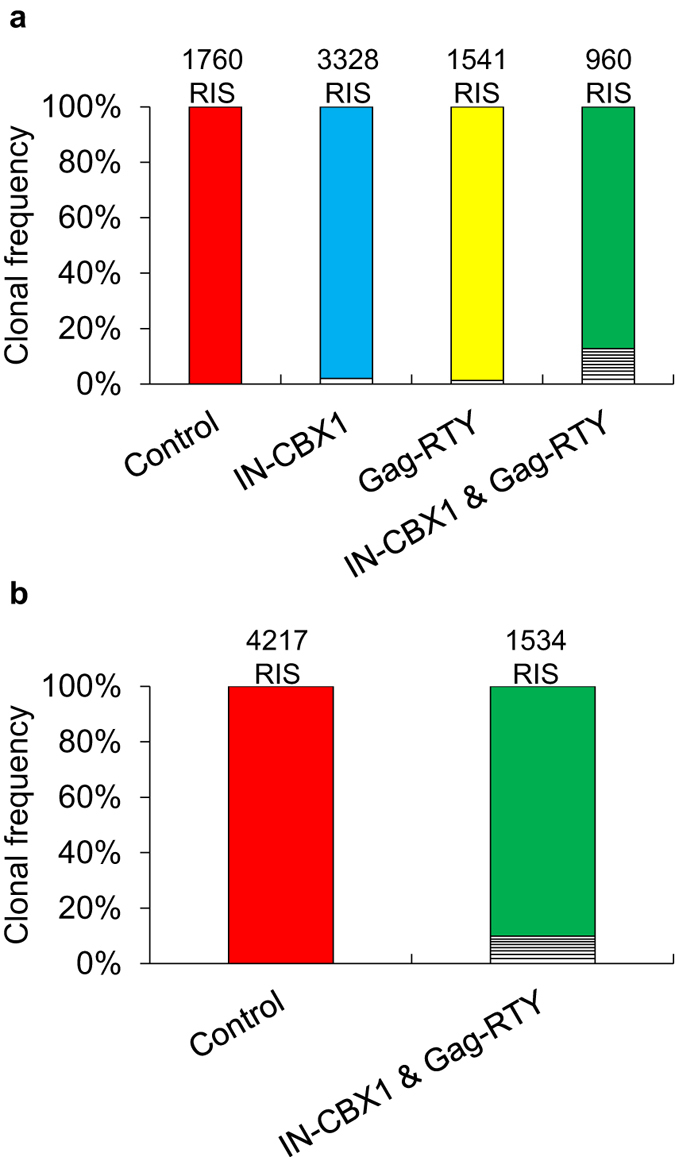
Retargeted FVs lead to highly polyclonal populations in IMR90 cells and CD34^+^ cells. (**a**) IMR90 cell populations. (**b**) CD34^+^ cell populations. Integration sites with a capture frequency ≥1% are represented by white boxes in ascending order. The total number of integration sites for the associated sample are listed on the top of each bar. RIS, retroviral vector integration sites.

Finally, we analyzed the clonal diversity of the transduced IMR90 and CD34^+^ cells by RIS analysis (Fig. 7 and Supplemental Table S2). We observed highly polyclonal populations with no evidence of dominant clones for all vectors in both cell types.

## Discussion

Gene therapy has enormous potential but genotoxicity is a major concern for use of retroviral vectors in the clinic. Here we have successfully modified the integration profile of FV, resulting in significantly fewer RISs near genes and proto-oncogenes which may prove to be safer. Retargeted FVs can be produced by simply using the modified Gag and Pol helper plasmids we describe here during vector production. Retargeted FVs can be produced at high titer and efficiently transduce human CD34^+^ cells suggesting they will be useful for gene therapy of SCID-X1, chronic granulomatous disease, thalassemias, and potentially metabolic diseases that can be treated by hematopoietic stem cell (HSC) transplantation such as metachromatic leukodystrophy.

Of critical importance for gene therapy is the effect of modifications, such as protein fusions and amino acid substitutions, on vector titer, transduction efficiency, and transgene expression. Although the mutations needed to retarget FV modestly reduced titers, we were able to obtain clinically relevant titers (>10^7^ TU/ml) after concentration. We also observed high levels of transgene expression relative to the control that were achieved using a low MOI in both normal human fibroblasts and cord blood CD34^+^ cells. There was also no evidence of vector silencing in CD34^+^ cells using the strong spleen focus forming virus (SFFV) promoter. The Gag-RTY modification reduced vector titer which is consistent with a study by Tobaly-Tapiero *et al.* in which they found fewer integrated viral copies in genomic DNA when the vector expressed Gag-RTY when compared to a control FV^23^. It was found Gag-RTY abolishes the affinity of FV PIC for host H2A and H2B histones, which reduces the efficiency of integration.

A surprising observation was the stronger influence of the Gag-RTY modification on RIS retargeting than the IN-CBX1 modification. Although it is known that the foamy retroviral Gag CBS interacts with H2A and H2B histones and is partially responsible for tethering the PIC to the host chromatin^23^, this is the first study that demonstrates mutations in the Gag CBS substantially retargets FV integration. FV_Gag-RTY_ integration frequently occurred in H3K9me3 regions (Fig. 3), which are known to be a preferred target for CBX1. Upon further analysis, it was determined that many of the retargeted RISs were also in satellite repeat elements and near centromeres. Satellite DNA is the main component of centromeres and heterochromatin. Maskell *et al.* demonstrated that the ability of the FV intasome to form a stable complex with host chromatin, a prerequisite for integration, is dependent on the ability to engage nucleosomes^26^. It was further shown that specific amino acid substitutions in IN impede the ability of the FV intasome to interact with nucleosomes. Interestingly, these IN mutant FVs integrated more frequently within genes, which is a stark contrast to the reduction of integration within genes observed in our study with FVs harboring Gag-RTY (Table 1). The loss of the CBS functionality may alter the genomic locations accessible to the FV PIC and this may be dependent on the local chromatin structure immediately preceding integration. There may also be viral or host proteins that interact with foamy viral Gag CBS that influence integration site preferences which have yet to be determined. Our findings suggest the Gag CBS has a major role in integration site selection and Gag-RTY retargets FV integration into satellite repeats which are predominately gene sparse and frequently bear the H3K9me3 mark.

The addition of IN-CBX1 significantly increased the number of RISs within H3K9me3 regions. We also observed that FV_IN-CBX1 & Gag-RTY_ had a greater preference for DNase I hypersensitivity, H3K9ac, and H3K4me3 sites than FV_Control_ (Supplemental Figure S2). A possible explanation is that vectors retargeted to H3K9me3 sites or satellite elements have a preference for chromatin that is more accessible. However, it is important to consider that the state of histone methylation and acetylation during integration is not known. It has been shown that certain genes transiently bear the H3K9me3 mark during transcription activation^27^. IMR90 cells transduced with FV_IN-CBX1 & Gag-RTY_ had a much greater number of RISs in satellites (Table 2) and did not have dramatically more RISs in H3K9ac regions (Supplemental Figure S2) when compared to FV_Control_, which suggests the retargeted FV is not being directed to H3K9me3 regions overlapping highly expressed genes. It is also important to determine if a retroviral vector has any strong integration site preferences and thus has a tendency to form integration hotspots. Hotspots were observed at much higher frequency with FV_IN-CBX1 & Gag-RTY_ than FV_Control_ (Fig. 4). However, FV_IN-CBX1 & Gag-RTY_ RISs in hotspots had a strong tendency to also be near H3K9me3 regions. FV_IN-CBX1 & Gag-RTY_ formed hotspots in putatively safe genomic regions, as H3K9me3 sites are associated with gene sparse regions. Also, we observed that RISs near H3K9me3 sites were farther away from proto-oncogene TSSs on average in CD34^+^ cells (Supplemental Table S1). Thus retargeted FVs have increased hotspots, but these are in gene sparse areas. This observation also provides more evidence that utilizing both the IN-CBX1 fusion protein and the Gag-RTY mutant protein results in the most efficient retargeting into gene sparse regions. The formation of integration hotspots thus appears to be a result of retargeted integration site selection rather than an indication of genotoxicity. Clonality analyses showed that all IMR90 and CD34^+^ cell populations were polyclonal at 23 days post vector exposure (DPVE) and 10 DPVE respectively. FV_IN-CBX1 & Gag-RTY_ had a slight decrease in the number of clones contributing to the total cell population, with 8 clones contributing 1–2% in CD34^+^ cells at 10 DPVE. However, it is important to note that all of the RISs were greater than 100 kb from the nearest proto-oncogene TSS in these clones. Also, the total number of clones contributing to the retargeted FV cell populations may be slightly underrepresented due to the increased number of RISs in repetitive regions which are not included in clonality analyses. Future *in vivo* studies are needed to determine if FV_IN-CBX1 & Gag-RTY_ has a dramatic effect on the resulting hematopoietic repopulating cell populations.

It has been shown that modifying the host cells to express a lentiviral IN-LEDGF fusion protein can alter the integration profile of LVs^20^. However, methods that require modifying the host cells prior to transduction are not a clinically relevant approach. As an alternative to manipulating tethering factors, proteins such as zinc finger and endonucleases fused directly to IN have shown weak retargeting in LVs^29^,^30^,^31^. Although there were only slight effects on retargeting integration, these experiments demonstrated the feasibility of modifying vectors directly. Recently, Ashkar *et al.* demonstrated GV integration can be targeted away from active transcription areas in the genome by introducing mutations in the IN regions that interact with bromodomain and extra-terminal (BET) proteins. GV IN interacts with host BET proteins and directs the GV PIC into or near TSSs^32^,^33^,^34^. Specifically in this study, single point mutations or complete truncation of the C-terminal region of GV IN resulted in an integration profile distinct from normal GV, with fewer RISs being near proto-oncogene TSSs^35^. However, vector titers were not expressed in TU/ml and the distributions of RISs were not analyzed in human cord blood CD34^+^ cells in their study. Also, while it was shown that the modified GVs had a similar percentage of RISs near proto-oncogene TSSs when compared to published normal FV data, we have demonstrated that the retargeted FVs have even less of a preference for proto-oncogene TSSs than normal FVs (Table 1). Thus, retargeted FV have a potentially safer integration profile than BET retargeted GV. Other inherent advantages of using FVs over other retroviral vectors include a lower potential to activate nearby genes and they have not been shown to exhibit read-through transcription^12^,^22^.

An important advantage of our approach is the ability to transduce target cells by simply using different helper plasmids during vector production. To obtain optimal retargeting of FV into H3K9me3 regions, both the Gag mutant and the IN-CBX1 fusion protein were necessary. This suggests that reducing the affinity of the foamy retroviral Gag CBS for H2A and H2B histones may be necessary to facilitate retargeting FVs with a modified IN. Importantly, we also show that the foamy IN can tolerate relatively large protein fusions. The CBX1 protein fused to the foamy IN is 21 kDa. Using Gag-RTY in conjunction with other FV IN fusion proteins, such as an IN-zinc finger or an IN-Cas9 fusion protein, should be considered in future work when developing retargeted FVs.

In summary the retargeted FVs we have described here integrate less frequently near proto-oncogenes than control FV. Since FV proviruses are also known to dysregulate nearby genes less than LVs or GVs^36^, retargeted FVs may be a safer option for HSC gene therapy. Thus, the safety of retargeted FVs should be further explored for clinical use.

## Materials and Methods

### Developing constructs and vector production

Vector FV-SGW-KO contains an SFFV promoter driving expression of EGFP. The FV helper plasmids pFVPol-CBX1 and pFVGag-CBS-RTY (Fig. 1) were constructed using standard molecular biology techniques. pFVPol-CBX1 was constructed by replacing the IN gene of pFVPolCO with the IN-CBX1 fusion gene. pFVGag-CBS-RTY was constructed by replacing the wild type Gag CBS domain of pFVGagCO with the RTY mutant CBS domain.

Vectors were produced by transient transfection of FV plasmid and helper plasmids on HEK-293 cells using polyethylenimine as previously described^37^, except that 14 μg of FV vector plasmid FV-SGW-KO and 0.3, 4, and 6 μg of the appropriate Env, Gag, and Pol FV helper plasmids were used, and 72.9 μg polyethylenimine were used and 4-(2-hydroxyethyl)-1-piperazineethanesulfonic acid was omitted. Vector-containing supernatant was passed through a 0.45 μm filter (Pall Corporation, Newquay, United Kingdom), concentrated 100-fold by ultracentrifugation at 23 °C, and frozen at −80 °C until use in serum-free media containing 5% DMSO. Before use on CD34^+^ human cord blood, vector preparations were dialyzed using an Amicon Ultra 0.5 mL Centrifugal 50 kDa Filter (EMD Millipore, Billerica, MA) to remove DMSO. Vector preparations were titered on HT1080 cells and EGFP expressing cells were quantified using a BD Accuri C6 Flow Cytometer 72 hours post vector exposure (BD Biosciences, Franklin Lakes, NJ).

### Cell culture, transduction of IMR90 and CD34^+^ human cord blood cells, and CFU assays

IMR90 cells (Coriell Institute, Camden, NJ) were cultured in Eagle’s minimal essential medium (EMEM) (ATCC, Manassas, VA) supplemented with 15% Fetal bovine serum (FBS) (Atlanta Biologicals, Lawrenceville, GA) at 37 °C in 5% CO_2_. For each vector, six 10-cm dishes were seeded with 5 × 10^4^ cells on day 1, the appropriate vector was added at a multiplicity of infection of 0.5 on day 2, the cells were passaged 1:3 to one 10 cm plate on day 7, the cells were expanded to two 10 cm plates on day 12, and the DNA was extracted on day 23.

Cryopreserved CD34^+^ male human cord blood cells (Stemcell Technologies, Vancouver, Canada) were thawed, counted, and plated in a 12-well tissue culture treated plate at 5 × 10^5^ cells/mL in prestimulation media (IMDM + 10% heat inactivated FBS with 5000U penicillin/streptomycin and cytokines: rhIL-3, rhIL-6, rhSCF, rhTPO, rhFlt-3, rhG-CSF (ProSpec-Tany TechnoGene Ltd., Rehovot, Israel), 100 ng/mL each) on day 1. Cells were incubated at 37 °C overnight. On day 2, a human fibronectin fragment coated 12-well suspension plate was prepared by coating wells with 2 μg/cm^2^ RetroNectin^®^ Reagent (Takara Bio, Otsu, Shiga, Japan). Cells were counted and plated in three wells at 5 × 10^5^ cells/well in prestimulation media. Cells were exposed to FV at an MOI of 10, or were mock transduced with prestimulation media. The final volume of each well was adjusted to 1 mL with prestimulation media and cells were incubated at 37 °C for 20 hours. On day 3, cells were washed, counted and plated at 5 × 10^5^ cells/ml. Cells were maintained between 5 × 10^5^ and 1 × 10^6^ cells/ml for the remainder of the experiment. DNA was extracted and the cells were analyzed for EGFP expression by flow cytometry on days 6 and 11. Events were viewed on a live cell gate for the flow cytometry analysis. One day after cells were exposed to vector, 2000 cells were plated in semisolid Methocult™ H4230 methylcellulose media (Stemcell Technologies, Vancouver, Canada). Methocult™ H4230 was prepared according to manufacturer’s directions, and the following cytokines were added: 50 ng/mL rhSCF, 20 ng/mL rhIL-3, 20 ng/mL rhG-CSF, 20 ng/mL rhGM-CSF (ProSpec-Tany TechnoGene Ltd., Rehovot, Israel). Methocult™ H4230 plates were incubated at 37 °C. 11 days later, CFUs were counted and scored for EGFP expression using fluorescence microscopy.

### Quantitative PCR for vector copy number analysis

Analysis of vector copy number was performed through a multiplexed quantitative PCR assay using primers and probes targeting an EGFP transgene and RNase P as an internal control. The EGFP/EYFP assay consisted of a Custom TaqMan Probe (#4316034) containing a 5′ FAM reporter dye and a 3′ Minor Groove Binder/nonfluorescent quencher, with primers/probe sequences as previously reported^38^. The RNase P assay (Applied Biosystems #4403328) contained a probe with a 5′ Hex reporter dye. The standard curve was generated with genomic DNA extracted from HT1080 cells transduced with a single vector containing EYFP. Reactions were run in triplicate with TaqMan Genotyping Master Mix (Applied Biosystems # 4371353) in a Bio-Rad CFX384 Touch under the following thermal cycling conditions: 95 °C 10 min + 40 × (95 °C 15 s + 60 °C 1 min).

### Analysis of integration sites in FV transduced cells

Genomic DNA was extracted from IMR90 normal human fibroblasts and CD34^+^ human cord blood cells using the Puregene Cell & Tissue kit (Qiagen Inc., Valencia, CA) according to the manufacturer’s directions. Vector-genome junctions were sequenced using MGS-PCR^25^.

Forward and reverse sequence reads were paired with PEAR software using the default settings^39^. Sequence reads were truncated at the 3^rd^ nucleotide letter that had a corresponding Q score < 27 (*p* ≥ 0.002) to reduce the effects of sequencing errors when aligning queries to the genome. Perl scripts were used to process and map vector-genome junctions to the Genome Reference Consortium (GRC) build GRCh37 (hg19) of the human genome^40^. Integration sites that aligned to repetitive regions in the genome that could not be clearly resolved as follows below were excluded from all downstream analyses, except for the analysis of the proximity of integration sites to repeat elements and centromeres. An integration site was considered to be within a repetitive region if its second greatest scoring alignment had an alignment score >95% than its greatest scoring alignment. For integration sites with alignment scores < 100, this threshold was reduced to 90%.

Perl scripts were used to determine the proximity of integration sites to RefSeq genes and TSSs, centromeres, repeat elements (LINE, SINE, LTR, DNA, and satellite elements), CpG islands, histone modification ChIP-Seq peaks, DNase I hypersensitive site peaks, and proto-oncogenes. RefSeq genes, repeat elements, centromere locations, and CpG islands files for the hg19 genome were downloaded from UCSC (http://genome.ucsc.edu/cgi-bin/hgTables). The Roadmap Epigenomics Project IMR90 and CD34^+^ histone modification ChIP-Seq and DNase I hypersensitive site peak files were used^41^. The annotated peaks for each cell type were sorted by peak strength and then divided into 10 equal sized bins, with bin 1 containing the lowest scoring peaks and bin 10 containing the highest scoring peaks (strong peaks). An integration site was considered to be within a peak if it was between the start and stop positions of a peak and an integration site was considered near a peak if it was within 50 kb of the start or stop site of a peak. For the cancer gene analysis, we used 2,048 cancer genes that had corresponding RefSeq mRNAs from the Network of Cancer Genes 4.0 as of June 18^th^, 2014. An integration site was considered to be within an oncogene when it was located within the oncogene transcript. RIS hotspots were defined as 3 RISs within the specified kb window. The RIS hotspot analysis was performed as previously described^42^. Briefly, RIS data sets were divided into a minimum of three non-overlapping randomly selected matched sized data sets of 320 RISs for IMR90 cell data and 511 RISs for CD34^+^ cell data for hotspot analyses.

### Statistical analysis

Statistical significance was determined by using Student’s *t*-test for vector titers and the χ^2^ goodness-of-fit test for all other analyses.

## Additional Information

**How to cite this article**: Hocum, J. D. *et al.* Retargeted Foamy Virus Vectors Integrate Less Frequently Near Proto-oncogenes. *Sci. Rep.* **6**, 36610; doi: 10.1038/srep36610 (2016).

**Publisher’s note:** Springer Nature remains neutral with regard to jurisdictional claims in published maps and institutional affiliations.

## Supplementary Material

Supplementary Information

## Figures and Tables

**Table 1:** Retargeted FVs integrate farther away from proto-oncogenes and other genomic features.

Vector	Total sites	Percent of RIS				
		In RefSeq genes	<10 kb from TSS	CpG islands^a^	<50 kb from proto- oncogene TSS	<500 kb from proto-oncogene TSS
Control_IMR90_	1760	36.6	25.2	9.7	3.6	23.5
IN-CBX1_IMR90_	3328	35.5	26.5	8.7	3.9	23.3
Gag-RTY_IMR90_	1541	32.3***	12.1***	2.4***	1.5***	13.4***
IN-CBX1 & Gag-RTY_IMR90_	960	27.5***^,^##	12.3***	3.0***	1.7***	13.5***
Control_CD34_	4217	40.9	24.5	6.5	4.7	23.3*
IN-CBX1 & Gag-RTY_CD34_	1534	30.6***	12.5***	2.5***	2.2***	15.3***
Random	10000	49.4	14.5	2.4	6.8	44.6

^***^Statistically significant at *p* < 0.001 compared to control vector.

^##^Statistically significant at *p* < 0.01 compared to Gag-RTY vector.

^a^Within or less than 1 kb from a CpG island. RIS, retroviral vector integration sites; TSS, transcription start site.

**Table 2:** Retargeted FVs integrate within satellite repeat elements.

Vector	Total sites	Percent of RIS					
		Centromere^a^	In repeat elements				
			LINE	SINE	Satellite	DNA	LTR
Control_IMR90_	2306	3.9	20.9	20.7	2.4	2.6	7.3
IN-CBX1_IMR90_	3994	4.7**	16.5***	18.4***	3.1**	2.8	8.6**
Gag-RTY_IMR90_	3382	37.0***	16.0***	13.1***	36.2***	1.5***	4.8***
IN-CBX1 & Gag-RTY_IMR90_	2224	37.1***	18.8*^,^###	15.7***^,^###	36.2***	1.1***	4.1***
Control_CD34_	5351	1.3	16.8	22.7	1.0	2.3	7.5
IN-CBX1 & Gag-RTY_CD34_	3175	26.8***	16.5	18.3***	31.1***	1.4***	5.7***
Random	10000	3.5	21.0	12.8	0.4	3.5	9.6

^**^Statistically significant at *p* < 0.01 compared to control vector.

^***^Statistically significant at *p* < 0.001 compared to control vector.

^###^Statistically significant at *p* < 0.001 compared to Gag-RTY vector.

^a^Less than 1 Mbp from centromere. RIS, retroviral vector integration sites.

## References

[b1] Naldini L. *Ex vivo* gene transfer and correction for cell-based therapies. Nat. Rev. Genet. 12, 301–315 (2011). 21445084 10.1038/nrg2985

[b2] Hacein-Bey-Abina S. *et al.* Efficacy of gene therapy for X-linked severe combined immunodeficiency. N. Engl. J. Med. 363, 355–364 (2010). 20660403 10.1056/NEJMoa1000164 PMC2957288

[b3] Cavazzana-Calvo M. *et al.* Gene therapy of human severe combined immunodeficiency (SCID)-X1 disease. Science 288, 669–672 (2000). 10784449 10.1126/science.288.5466.669

[b4] Aiuti A. *et al.* Gene therapy for immunodeficiency due to adenosine deaminase deficiency. N. Engl. J. Med. 360, 447–458 (2009). 19179314 10.1056/NEJMoa0805817

[b5] Hacein-Bey-Abina S. *et al.* LMO2-associated clonal T cell proliferation in two patients after gene therapy for SCID-X1. Science 302, 415–419 (2003). 14564000 10.1126/science.1088547

[b6] Fischer A., Hacein-Bey-Abina S. & Cavazzana-Calvo M. Gene therapy of primary T cell immunodeficiencies. Gene 525, 170–173 (2013). 23583799 10.1016/j.gene.2013.03.092

[b7] Trobridge G. D. Genotoxicity of retroviral hematopoietic stem cell gene therapy. Expert Opin. Biol. Ther. 11, 581–593 (2011). 21375467 10.1517/14712598.2011.562496 PMC3443588

[b8] Touzot F. *et al.* Faster T-cell development following gene therapy compared with haploidentical HSCT in the treatment of SCID-X1. Blood 125, 3563–3569, 10.1182/blood-2014-12-616003 (2015). 25869287

[b9] Mitchell R. S. *et al.* Retroviral DNA integration: ASLV, HIV, and MLV show distinct target site preferences. PLoS Biol. 2, E234, 10.1371/journal.pbio.0020234 (2004). 15314653 PMC509299

[b10] Cattoglio C. *et al.* Hot spots of retroviral integration in human CD34+ hematopoietic cells. Blood 110, 1770–1778, 10.1182/blood-2007-01-068759 (2007). 17507662

[b11] Deichmann A. *et al.* Insertion sites in engrafted cells cluster within a limited repertoire of genomic areas after gammaretroviral vector gene therapy. Mol. Ther. 19, 2031–2039 (2011). 21862999 10.1038/mt.2011.178 PMC3222531

[b12] Trobridge G. D. *et al.* Foamy virus vector integration sites in normal human cells. Proc. Natl. Acad. Sci. USA 103, 1498–1503, 10.1073/pnas.0510046103 (2006). 16428288 PMC1360565

[b13] Montini E. *et al.* The genotoxic potential of retroviral vectors is strongly modulated by vector design and integration site selection in a mouse model of HSC gene therapy. J. Clin. Invest. 119, 964–975 (2009). 19307726 10.1172/JCI37630 PMC2662564

[b14] Matreyek K. A. & Engelman A. In Viruses Vol. 5, 2483–2511 (2013). 24103892 10.3390/v5102483 PMC3814599

[b15] Llano M. *et al.* LEDGF/p75 determines cellular trafficking of diverse lentiviral but not murine oncoretroviral integrase proteins and is a component of functional lentiviral preintegration complexes. J. Virol. 78, 9524–9537 (2004). 15308744 10.1128/JVI.78.17.9524-9537.2004 PMC506940

[b16] Lewinski M. K. *et al.* Retroviral DNA integration: viral and cellular determinants of target-site selection. PLoS Pathog. 2, e60, 10.1371/journal.ppat.0020060 (2006). 16789841 PMC1480600

[b17] Cherepanov P. *et al.* HIV-1 integrase forms stable tetramers and associates with LEDGF/p75 protein in human cells. J. Biol. Chem. 278, 372–381 (2003). 12407101 10.1074/jbc.M209278200

[b18] Meehan A. M. & Poeschla E. M. Chromatin tethering and retroviral integration: recent discoveries and parallels with DNA viruses. Biochim. Biophys. Acta. 1799, 182–191 (2010). 19836475 10.1016/j.bbagrm.2009.10.001 PMC7326329

[b19] Christ F. & Debyser Z. The LEDGF/p75 integrase interaction, a novel target for anti-HIV therapy. Virology 435 (2013). 10.1016/j.virol.2012.09.033 23217620

[b20] Gijsbers R. *et al.* LEDGF hybrids efficiently retarget lentiviral integration into heterochromatin. Mol. Ther. 18, 552–560 (2010). 20195265 10.1038/mt.2010.36 PMC2839429

[b21] Bannister A. J. *et al.* Selective recognition of methylated lysine 9 on histone H3 by the HP1 chromo domain. Nature 410, 120–124 (2001). 11242054 10.1038/35065138

[b22] Trobridge G. D. Foamy virus vectors for gene transfer. Expert Opin. Biol. Ther. 9, 1427–1436 (2009). 19743892 10.1517/14712590903246388 PMC2782412

[b23] Tobaly-Tapiero J. *et al.* Chromatin tethering of incoming foamy virus by the structural Gag protein. Traffic 9, 1717–1727 (2008). 18627573 10.1111/j.1600-0854.2008.00792.x

[b24] Beard B. C., Adair J. E., Trobridge G. D. & Kiem H. P. High-throughput genomic mapping of vector integration sites in gene therapy studies. Methods Mol. Biol. 1185, 321–344 (2014). 25062639 10.1007/978-1-4939-1133-2_22

[b25] Rae D. T., Collins C. P., Hocum J. D., Browning D. L. & Trobridge G. D. Modified Genomic Sequencing PCR Using the MiSeq Platform to Identify Retroviral Integration Sites. Hum. Gene Ther. Methods, 26, 221–227 (2015). 26415022 10.1089/hgtb.2015.060 PMC4677540

[b26] Maskell D. P. *et al.* Structural basis for retroviral integration into nucleosomes. Nature 523, 366–369 (2015). 26061770 10.1038/nature14495 PMC4530500

[b27] Vakoc C. R., Mandat S. A., Olenchock B. A. & Blobel G. A. Histone H3 lysine 9 methylation and HP1gamma are associated with transcription elongation through mammalian chromatin. Mol. Cell 19, 381–391 (2005). 16061184 10.1016/j.molcel.2005.06.011

[b28] Wiencke J. K., Zheng S., Morrison Z. & Yeh R. F. Differentially expressed genes are marked by histone 3 lysine 9 trimethylation in human cancer cells. Oncogene 27, 2412–2421 (2008). 17968314 10.1038/sj.onc.1210895

[b29] Bushman F. D. & Miller M. D. Tethering human immunodeficiency virus type 1 preintegration complexes to target DNA promotes integration at nearby sites. J. Virol. 71, 458–464 (1997). 8985371 10.1128/jvi.71.1.458-464.1997 PMC191072

[b30] Tan W., Zhu K., Segal D. J., Barbas C. F. 3rd & Chow S. A. Fusion proteins consisting of human immunodeficiency virus type 1 integrase and the designed polydactyl zinc finger protein E2C direct integration of viral DNA into specific sites. J. Virol. 78, 1301–1313 (2004). 14722285 10.1128/JVI.78.3.1301-1313.2004 PMC321411

[b31] Schenkwein D. *et al.* rDNA-directed integration by an HIV-1 integrase–I-PpoI fusion protein. Nucleic Acids Res. 41, e61, 10.1093/nar/gks1438 (2013). 23275537 PMC3597653

[b32] De Rijck J. *et al.* The BET family of proteins targets moloney murine leukemia virus integration near transcription start sites. Cell Rep. 5, 886–894, 10.1016/j.celrep.2013.09.040 (2013). 24183673 PMC4197836

[b33] Sharma A. *et al.* BET proteins promote efficient murine leukemia virus integration at transcription start sites. Proc. Natl. Acad. Sci. USA 110, 12036–12041, 10.1073/pnas.1307157110 (2013). 23818621 PMC3718171

[b34] Gupta S. S. *et al.* Bromo- and extraterminal domain chromatin regulators serve as cofactors for murine leukemia virus integration. J. Virol. 87, 12721–12736 (2013). 24049186 10.1128/JVI.01942-13 PMC3838128

[b35] El Ashkar S. *et al.* BET-independent MLV-based Vectors Target Away From Promoters and Regulatory Elements. Mol. Ther. Nucleic Acids 3, e179, 10.1038/mtna.2014.33 (2014). 25072693 PMC4121521

[b36] Hendrie P. C., Huo Y., Stolitenko R. B. & Russell D. W. A Rapid and Quantitative Assay for Measuring Neighboring Gene Activation by Vector Proviruses. Mol. Ther. 16, 534–540 (2008). 18209733 10.1038/sj.mt.6300398

[b37] Kiem H. P. *et al.* Foamy combinatorial anti-HIV vectors with MGMTP140K potently inhibit HIV-1 and SHIV replication and mediate selection *in vivo*. Gene Ther. 17, 37–49 (2010). 19741733 10.1038/gt.2009.118 PMC3162371

[b38] Zhou S. *et al.* A self-inactivating lentiviral vector for SCID-X1 gene therapy that does not activate LMO2 expression in human T cells. Blood 116, 900–908, 10.1182/blood-2009-10-250209 (2010). 20457870 PMC2924228

[b39] Zhang J., Kobert K., Flouri T. & Stamatakis A. PEAR: a fast and accurate Illumina Paired-End reAd mergeR. Bioinformatics 30, 614–620 (2014). 24142950 10.1093/bioinformatics/btt593 PMC3933873

[b40] Hocum J. D. *et al.* VISA-Vector Integration Site Analysis server: a web-based server to rapidly identify retroviral integration sites from next-generation sequencing. BMC Bioinf. 16, 212, 10.1186/s12859-015-0653-6 (2015). PMC4493804 26150117

[b41] Bernstein B. E. *et al.* The NIH Roadmap Epigenomics Mapping Consortium. Nat. Biotechnol. 28, 1045–1048 (2010). 20944595 10.1038/nbt1010-1045 PMC3607281

[b42] Browning D. L. *et al.* Insulated Foamy Viral Vectors. Hum. Gene Ther. 27, 255–266 (2015). 10.1089/hum.2015.110 PMC4800274 26715244

