# Supplementary material for: Retargeted Foamy Virus Vectors Integrate Less Frequently Near Proto-oncogenes
Source: Sci Rep. 2016 Nov 4;6:36610. doi: 10.1038/srep36610 (PMC5095648; doi:10.1038/srep36610)
Supplement: Supplementary Information [file srep36610-s1.pdf]

Supplementary information:

Retargeted Foamy Virus Vectors Integrate Less Frequently Near Proto-oncogenes

Jonah D. Hocum [1], Ian Linde [1], Dustin T. Rae [1], Casey P. Collins [1], Lindsay K. Matern [1], and Grant D. Trobridge [1,2]

[1]Department of Pharmaceutical Sciences, Washington State University, Spokane, WA, United Sates, 99210; [2]School of Molecular Biosciences, Washington State University, Pullman, WA, United States, 99164

Figure S1

a

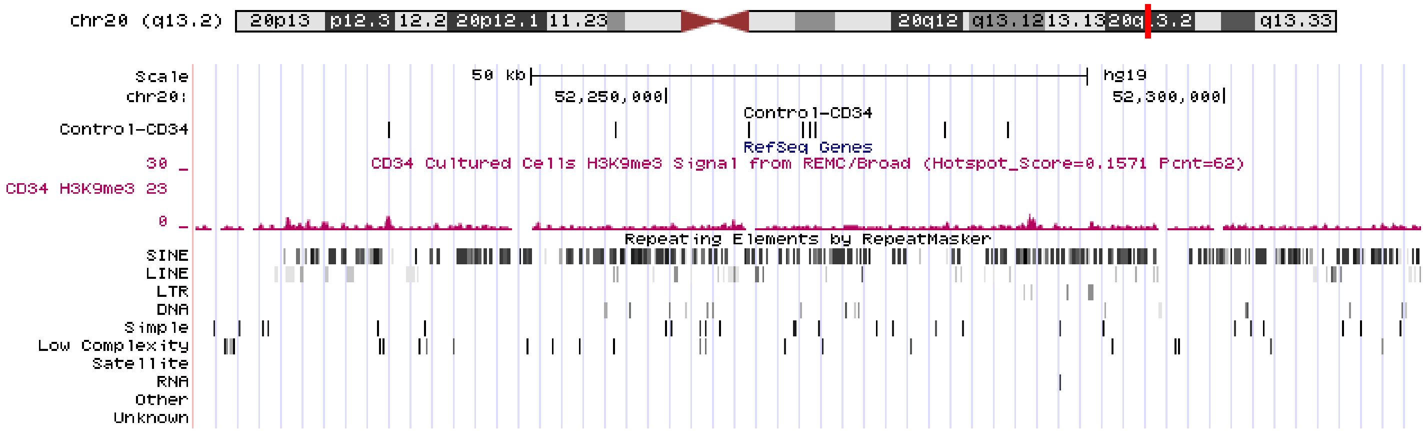

b

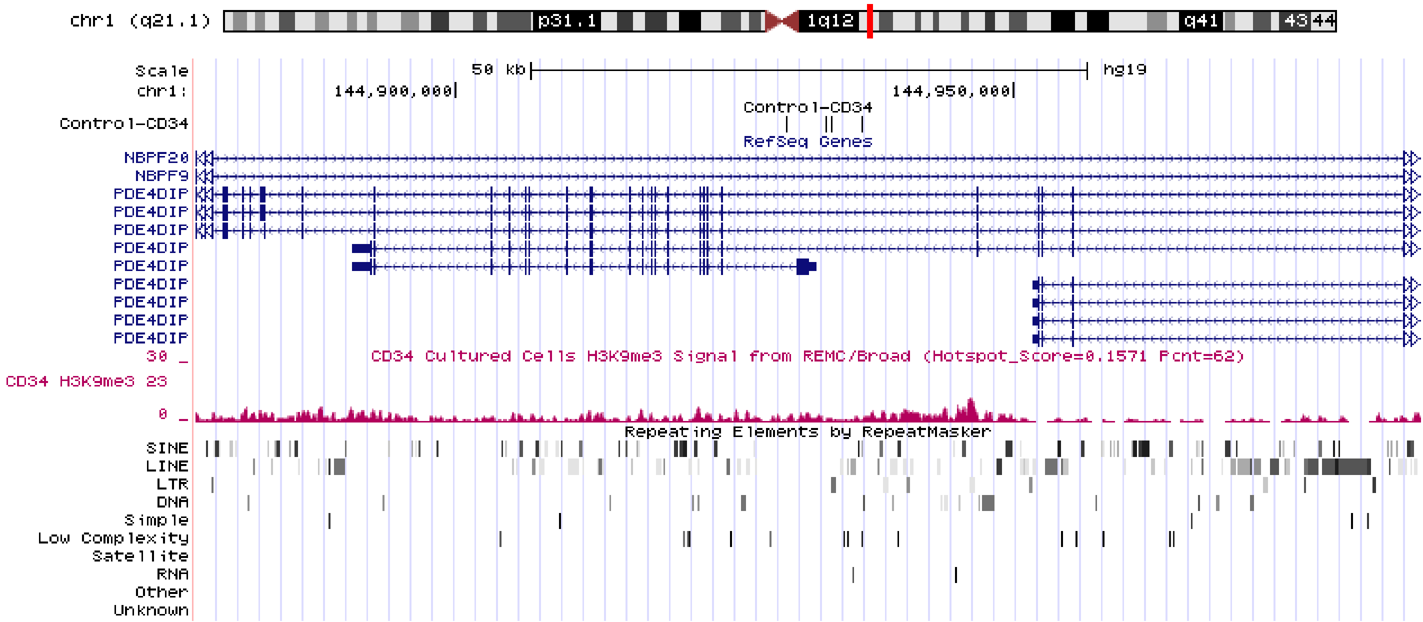

c

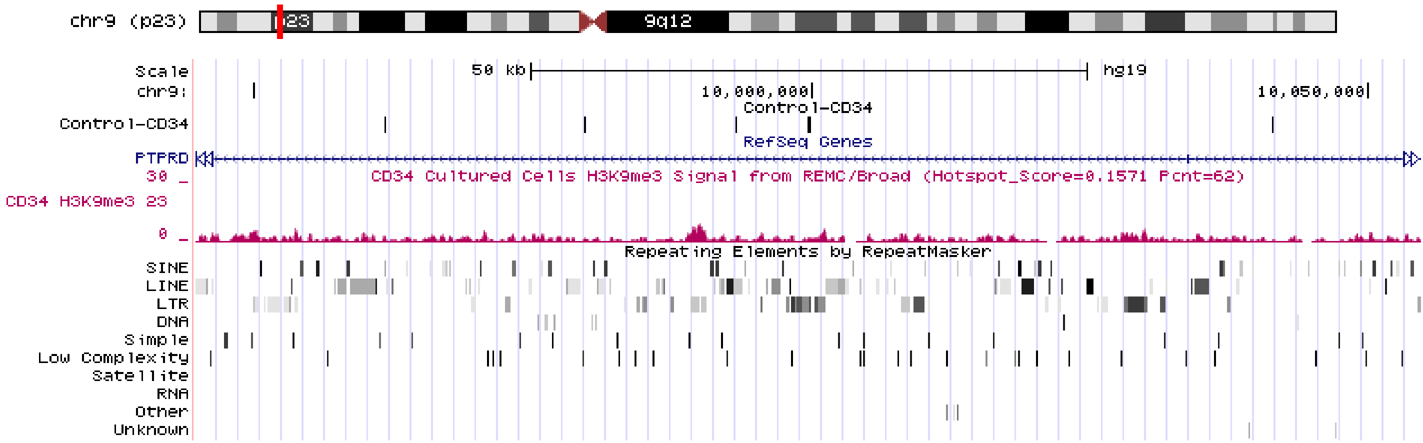

d

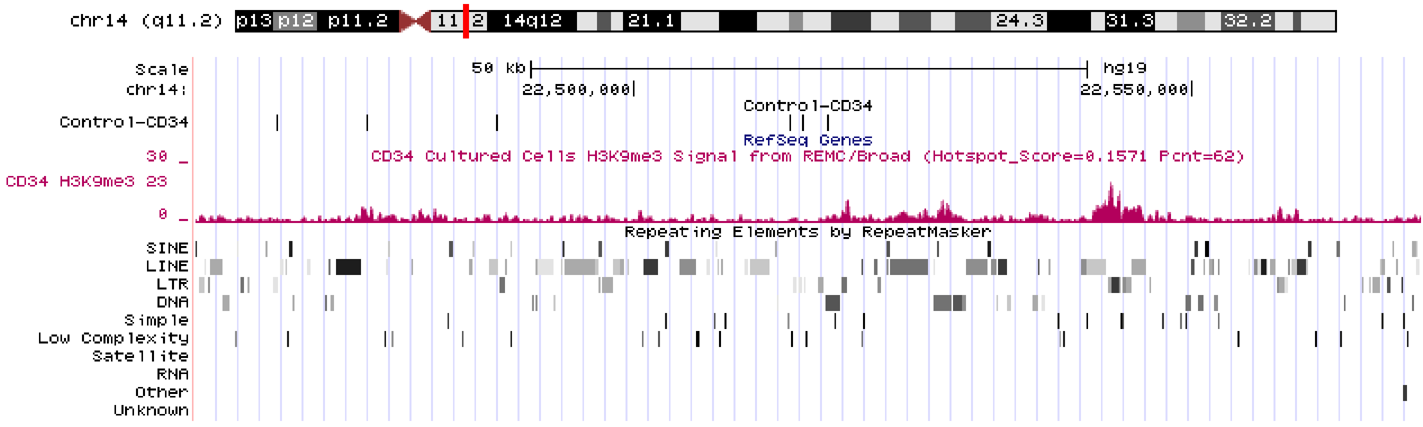

chr14 (q11.2)
p13 p12 p11.2 11 2 14q12 21.1 24.3 31.3 32.2
Scale
chr14:
50 kb
hg19
22,500,000|
22,550,000|
Control-CD34
Control-CD34
RefSeq Genes
30 _
CD34 Cultured Cells H3K9me3 Signal from REMC/Broad (Hotspot_Score=0.1571 Pcnt=62)
CD34 H3K9me3 23
0 _
Repeating Elements by RepeatMasker
SINE
LINE
LTR
DNA
Simple
Low Complexity
Satellite
RNA
Other
Unknown

e

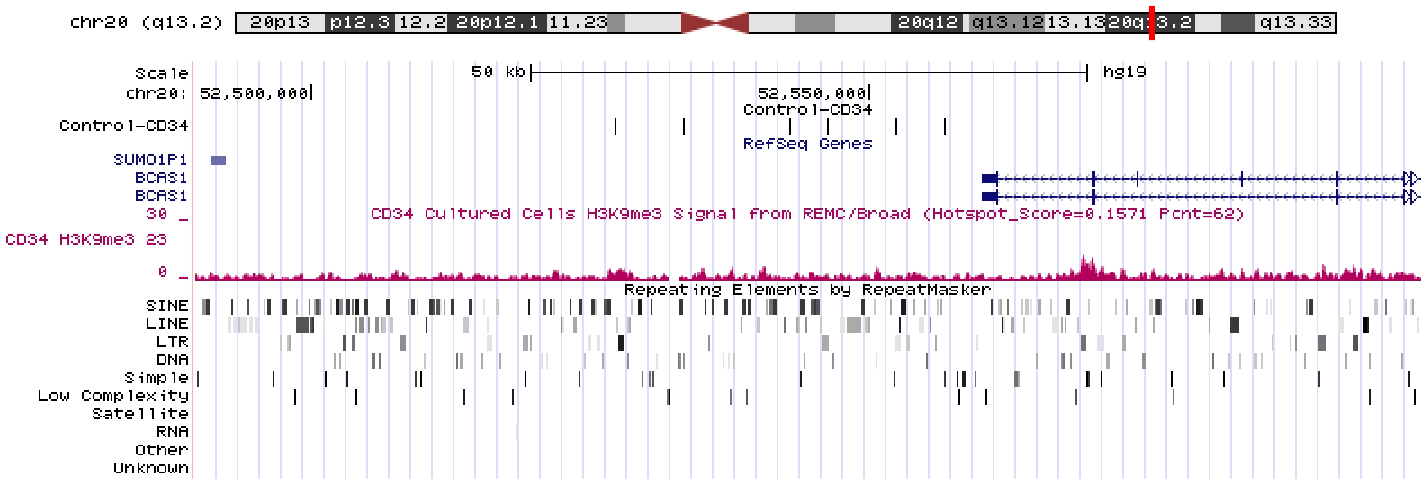

chr20 (q13.2)
20p13 p12.3 12.2 20p12.1 11.23 20q12 q13.12 13.13 20q 3.2 q13.33
Scale
chr20:
50 kb
hg19
52,500,000|
52,550,000|
Control-CD34
Control-CD34
RefSeq Genes
SUMO1P1
BCAS1
BCAS1
30 _
CD34 Cultured Cells H3K9me3 Signal from REMC/Broad (Hotspot_Score=0.1571 Pcnt=62)
CD34 H3K9me3 23
0 _
Repeating Elements by RepeatMasker
SINE
LINE
LTR
DNA
Simple
Low Complexity
Satellite
RNA
Other
Unknown

f

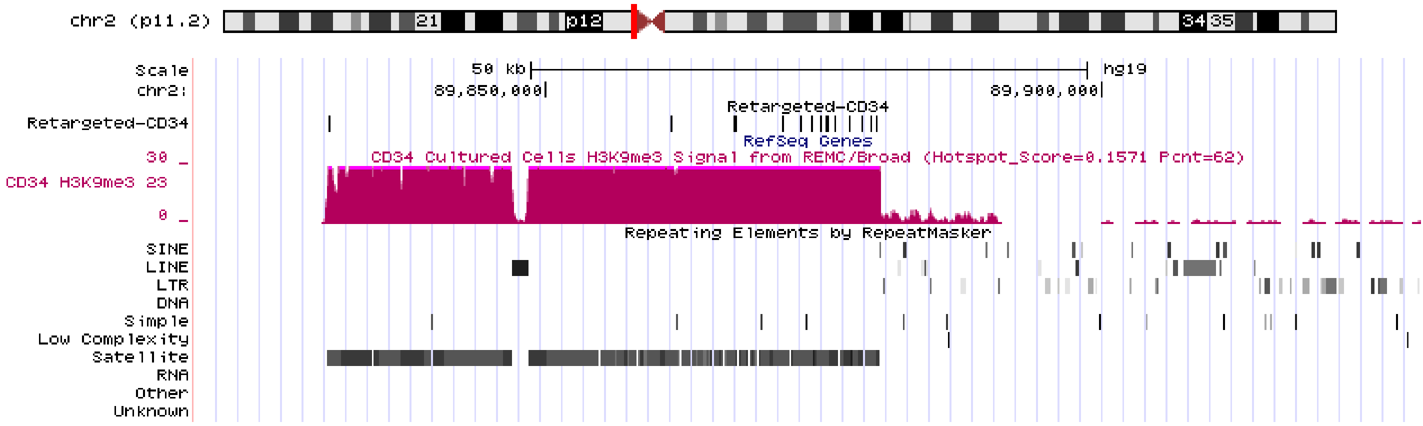
chr2 (p11.2)
21
p12
34
35
Scale
chr2:
50 kb
hg19
89,850,000
89,900,000
Retargeted-CD34
RefSeq Genes
30 _
CD34 H3K9me3 23
0 _
CD34 Cultured Cells H3K9me3 Signal from REMC/Broad (Hotspot_Score=0.1571 Pcnt=62)
Repeating Elements by RepeatMasker
SINE
LINE
LTR
DNA
Simple
Low Complexity
Satellite
RNA
Other
Unknown

g

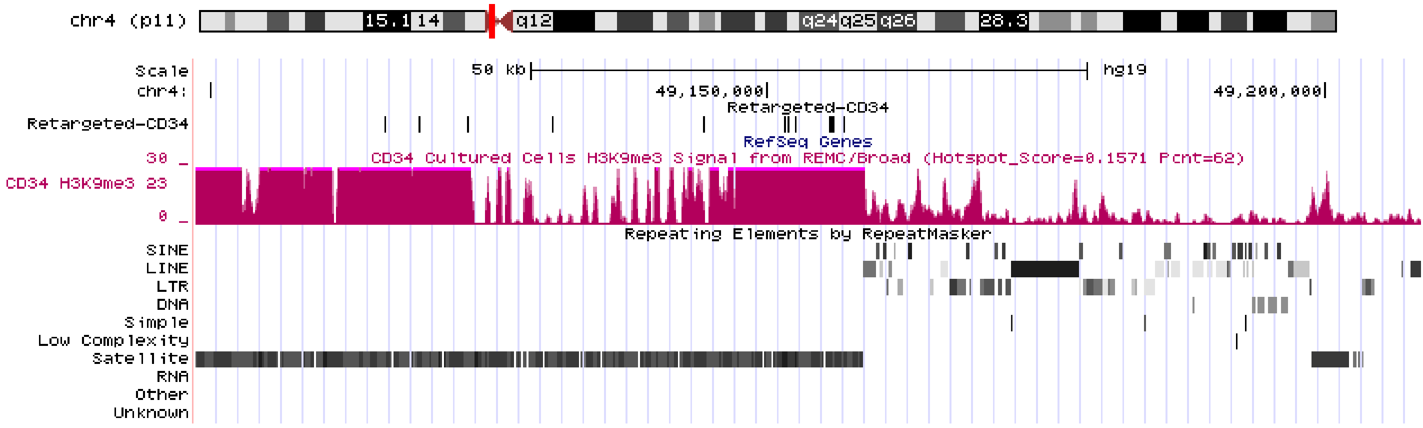
chr4 (p11)
15.1
14
q12
q24
q25
q26
28.3
Scale
chr4:
50 kb
hg19
49,150,000
49,200,000
Retargeted-CD34
RefSeq Genes
30 _
CD34 H3K9me3 23
0 _
CD34 Cultured Cells H3K9me3 Signal from REMC/Broad (Hotspot_Score=0.1571 Pcnt=62)
Repeating Elements by RepeatMasker
SINE
LINE
LTR
DNA
Simple
Low Complexity
Satellite
RNA
Other
Unknown

h

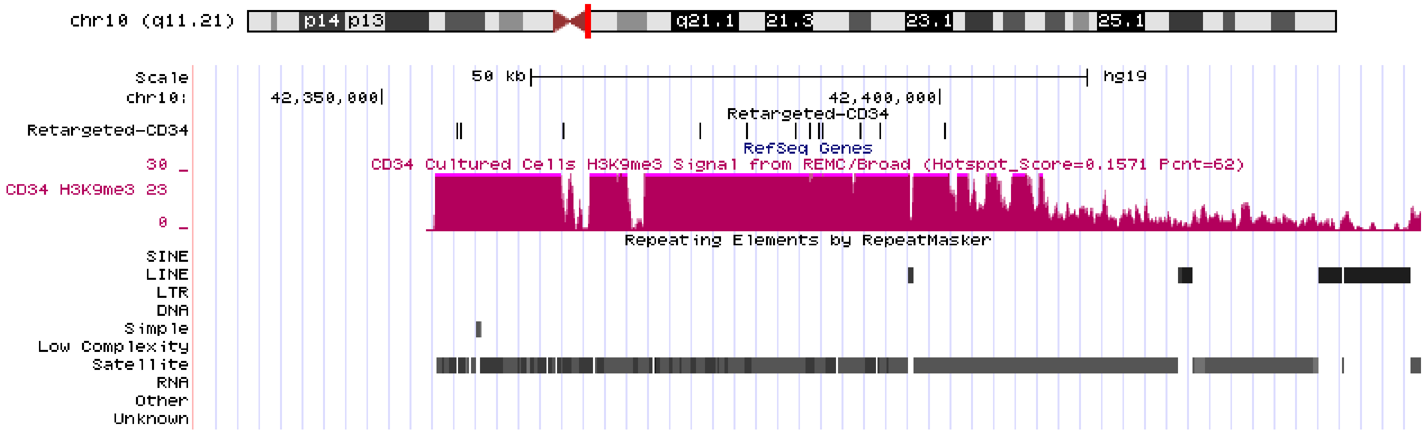
chr10 (q11.21)
p14
p13
q21.1
21.3
23.1
25.1
Scale
chr10:
50 kb
hg19
42,350,000
42,400,000
Retargeted-CD34
RefSeq Genes
30 _
CD34 H3K9me3 23
0 _
CD34 Cultured Cells H3K9me3 Signal from REMC/Broad (Hotspot_Score=0.1571 Pcnt=62)
Repeating Elements by RepeatMasker
SINE
LINE
LTR
DNA
Simple
Low Complexity
Satellite
RNA
Other
Unknown

i

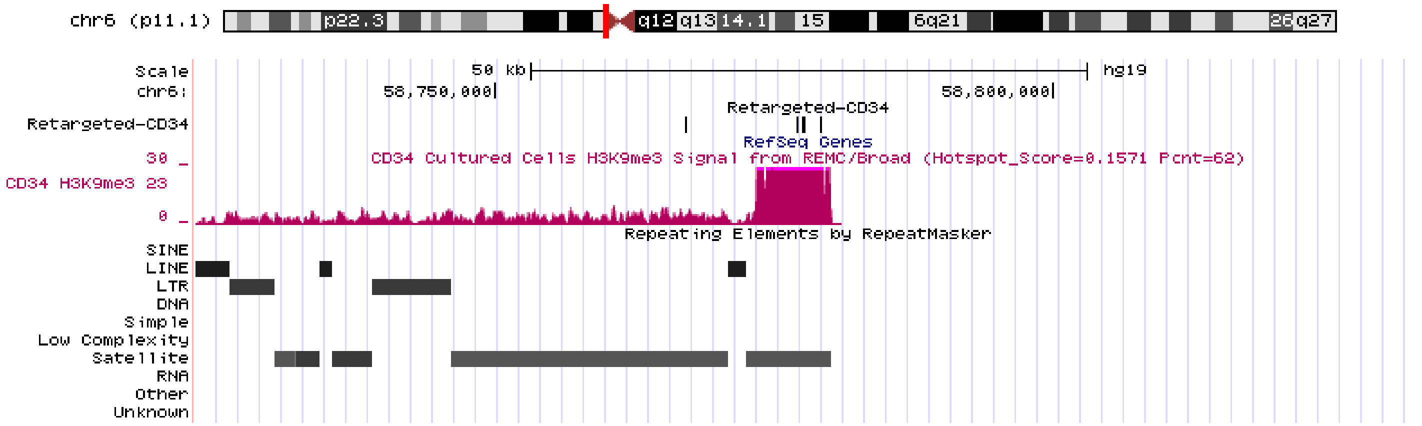

j

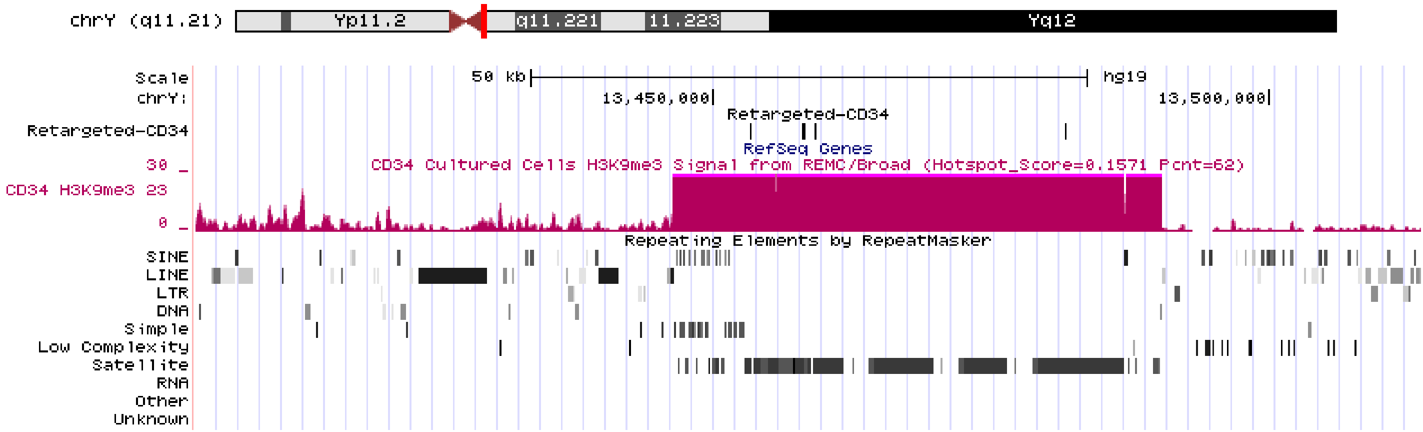

Figure S1. Example hotspots with RISs nearest to each other observed in CD34⁺ cells. Plotted in the UCSC genome browser. **(a-e)** Control FV example hotspots. **(f-j)** Retargeted FV example hotspots. The following UCSC genome browser tracks are also displayed: RefSeq Genes, CD34 Cultured Cells H3K9me3 Signal from REMC/Broad, and RepeatMasker. RIS, retroviral vector integration site.

Figure S2

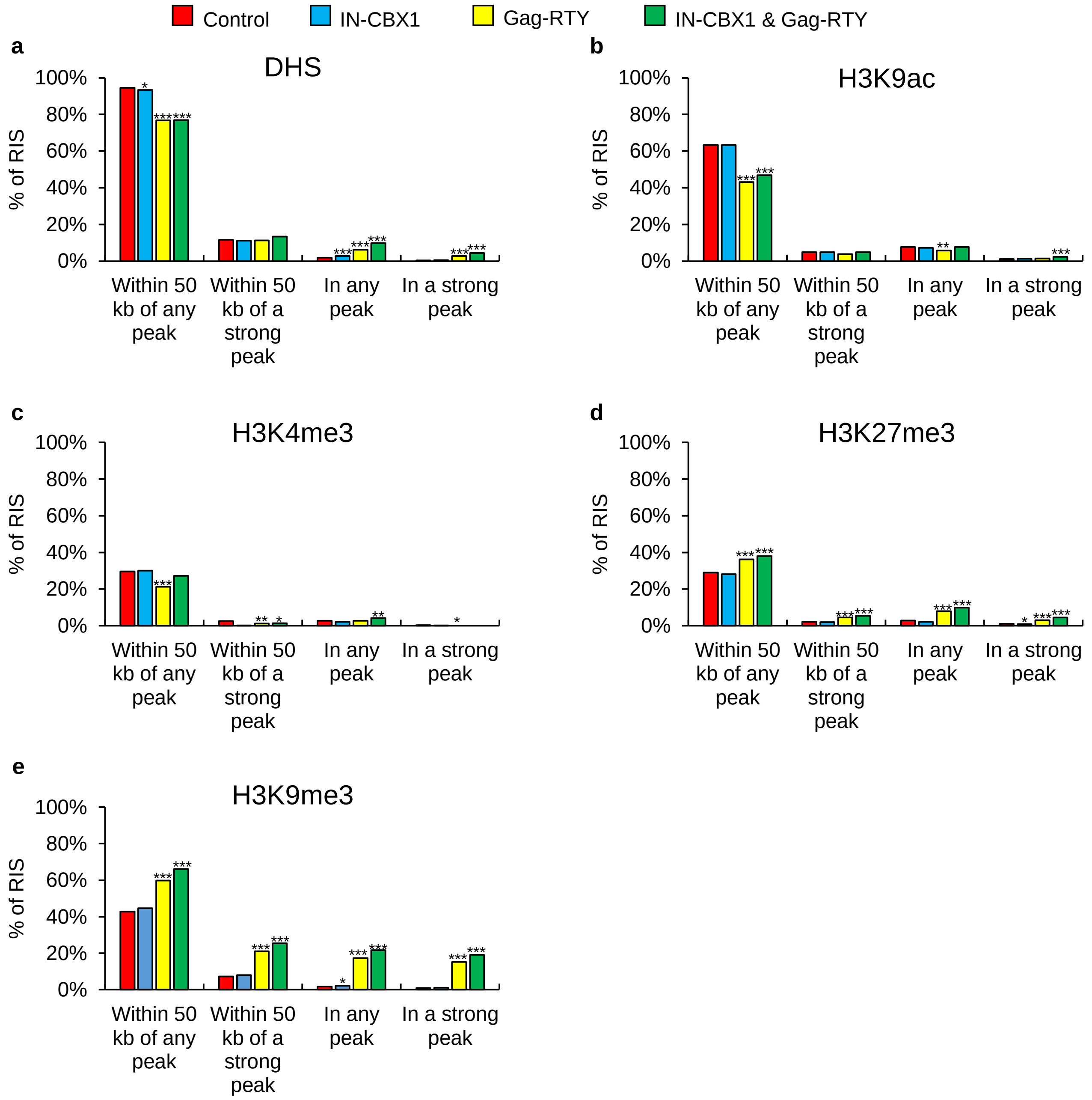

Figure S2. Proximity of FV integration sites in IMR90 cells to (a) DHS, (b) H3K9ac, (c) H3K4me3, (d) H3K27me3, and (e) H3K9me3. H3K9ac and H3K4me3 are associated with active genes and H3K27me3 is associated with inactive genes. **(e)** Is a duplicate representation of the IMR90 data from Figure 3a. DHS, DNase I hypersensitive sites; H3K9ac, acetylated lysine 9 of histone H3 sites; H3K4me3, tri-methylated lysine 4 of H3 histone sites; H3K27me3, tri-methylated lysine 27 of H3 histone sites; H3K9me3, tri-methylated lysine 9 of histone H3 sites. RIS, retroviral vector integration sites.

* statistically significant at $p < 0.05$ compared to control vector.

** statistically significant at $p < 0.01$ compared to control vector.

*** statistically significant at $p < 0.001$ compared to control vector.

Figure S3

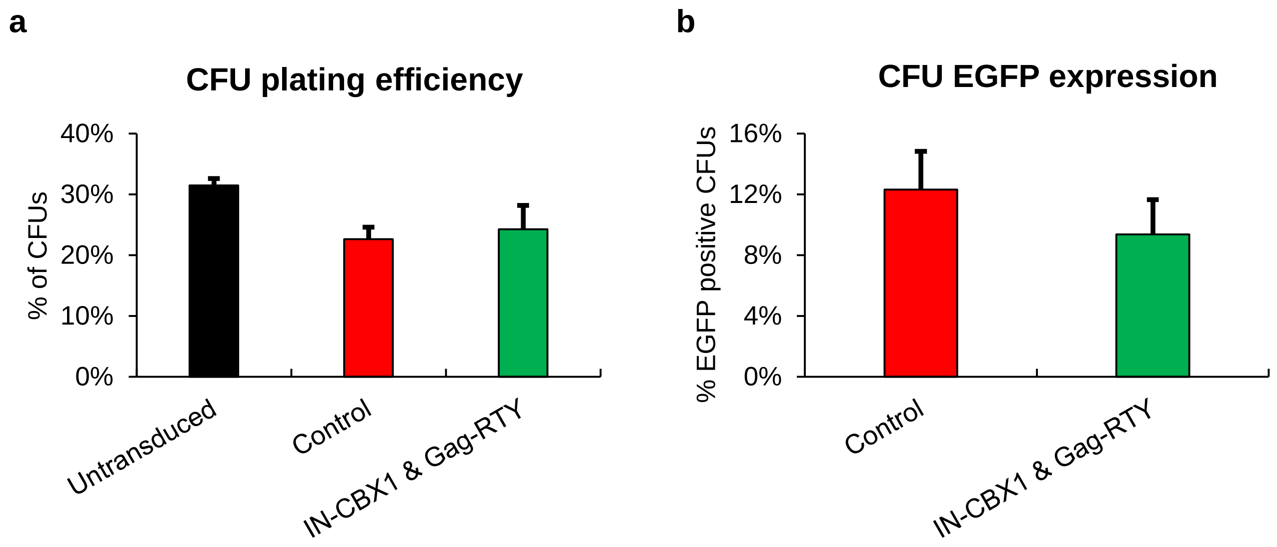

Figure S3. Efficient transduction of human CD34+ colony forming units. 2000 cells from each sample were plated in triplicate in Methocult™ H4230. A mean of 453 colonies were counted for the control FV group and a mean of 486 colonies were counted for the retargeted FV group. (a) Plating efficiency was calculated as the number of CFUs divided by the number of cells originally plated (mean + SEM). (b) EGFP positive CFUs were determined by fluorescence microscopy (mean + SEM). CFU; colony forming unit**.**

Table S1. Retargeted FV RIS near highly enriched H3K9me3 regions are farther away from proto-oncogene TSS.

	% < 50 kb from proto-oncogene TSS	% < 500 kb from proto-oncogene TSS	Mean distance	Standard deviation
**RIS < 50 kb from strong H3K9me3 peaks**	0.49 %	3.66 %	4.33 Mbp	6.62 Mbp
**RIS ≥ 50 kb from strong H3K9me3 peaks**	2.85 %	19.48 %	3.41 Mbp	4.35 Mbp

RIS, retroviral vector integration sites; TSS, transcription start sites.

Table S2. Clones constituting 1 % or more of the total capture frequency.

Vector	Cell line	Locus	Capture frequency	Within a strong H3K9me3 peak?	Within a satellite repeat?	Distance to nearest RefSeq TSS	Distance to nearest proto-oncogene TSS
IN-CBX1	IMR90	chr7:43058805	1.98 %	No	No	87 kb, MRPL32	1306 kb, CAMK2B

Vector	Cell line	Locus	Capture frequency	Within a strong H3K9me3 peak?	Within a satellite repeat?	Distance to nearest RefSeq TSS	Distance to nearest proto-oncogene TSS
Gag-RTY	IMR90	chr21:25198348	1.33 %	No	No	1606 kb, NCRNA00158	7733 kb, TIAM1

Vector	Cell line	Locus	Capture frequency	Within a strong H3K9me3 peak?	Within a satellite repeat?	Distance to nearest RefSeq TSS	Distance to nearest proto-oncogene TSS
IN-CBX1 & Gag-RTY	IMR90	chr16:34372309	1.73 %	No	No	32 kb, UBE2MP1	3006 kb, ITGAX
IN-CBX1 & Gag-RTY	IMR90	chr6:58776968	1.56 %	Yes	Yes	489 kb, GUSBL2	2269 kb, DST
IN-CBX1 & Gag-RTY	IMR90	chr5:62739196	1.34 %	No	No	518 kb, HTR1A	3857 kb, PDE4D
IN-CBX1 & Gag-RTY	IMR90	chr21:11115641	1.21 %	Yes	No	17 kb, BAGE	21816 kb, TIAM1
IN-CBX1 & Gag-RTY	IMR90	chrX:121344342	1.19 %	No	No	974 kb, GRIA3	2517 kb, SEPT6
IN-CBX1 & Gag-RTY	IMR90	chrX:130396526	1.18 %	No	No	27 kb, IGSF1	1152 kb, ELF4
IN-CBX1 & Gag-RTY	IMR90	chr4:53771317	1.15 %	No	No	43 kb, RASL11B	473 kb, FIP1L1
IN-CBX1 & Gag-RTY	IMR90	chrX:7856849	1.14 %	No	No	39 kb, PNPLA4	2732 kb, MID1
IN-CBX1 & Gag-RTY	IMR90	chr9:23671123	1.13 %	No	No	155 kb, ELAVL2	1677 kb, CDKN2A
IN-CBX1 & Gag-RTY	IMR90	chr3:76674593	1.06 %	No	No	415 kb, ROBO2	15437 kb, FHIT

Vector	Cell line	Locus	Capture frequency	Within a strong H3K9me3 peak?	Within a satellite repeat?	Distance to nearest RefSeq TSS	Distance to nearest proto-oncogene TSS
IN-CBX1 & Gag-RTY	$CD34^+$	chr2:43257083	1.83 %	No	No	197 kb, ZFP36L2	197 kb, ZFP36L2
IN-CBX1 & Gag-RTY	$CD34^+$	chr5:101508953	1.47 %	No	No	123 kb, SLCO4C1	5238 kb, LNPEP
IN-CBX1 & Gag-RTY	$CD34^+$	chr5:6838298	1.27 %	No	No	10 kb, MIR4278	5066 kb, CTNND2
IN-CBX1 & Gag-RTY	$CD34^+$	chr19:19165003	1.18 %	No	No	10 kb, SLC25A42	138 kb, LOC729991-MEF2B
IN-CBX1 & Gag-RTY	$CD34^+$	chr4:39172326	1.07 %	No	No	12 kb, WDR19	507 kb, KLF3
IN-CBX1 & Gag-RTY	$CD34^+$	chr9:97397671	1.03 %	No	No	5 kb, FBP1	682 kb, FANCC
IN-CBX1 & Gag-RTY	$CD34^+$	chr2:221480576	1.03 %	No	No	709 kb, MIR4268	1630 kb, FEV
IN-CBX1 & Gag-RTY	$CD34^+$	chr1:4850226	1.03 %	No	No	135 kb, AJAP1	1409 kb, RPL22

TSS, transcription start site.
